# Chimeric Translation for Mitochondrial Peptides: Regular and Expanded Codons

**DOI:** 10.1016/j.csbj.2019.08.006

**Published:** 2019-08-23

**Authors:** Hervé Seligmann, Ganesh Warthi

**Affiliations:** a The National Natural History Collections, The Hebrew University of Jerusalem, 91404 Jerusalem, Israel; b Aix-Marseille University, IRD, VITROME, Institut Hospitalo-Universitaire Méditerranée-Infection, Marseille, France

**Keywords:** delRNAs, Non-canonical translation, Non-canonical transcription, RNA editing, tRNA hopping

## Abstract

Frameshifting protein translation occasionally results from insertion of amino acids at isolated mono- or dinucleotide-expanded codons by tRNAs with expanded anticodons. Previous analyses of two different types of human mitochondrial MS proteomic data (Fisher and Waters technologies) detect peptides entirely corresponding to expanded codon translation. Here, these proteomic data are reanalyzed searching for peptides consisting of at least eight consecutive amino acids translated according to regular tricodons, and at least eight adjacent consecutive amino acids translated according to expanded codons. Both datasets include chimerically translated peptides (mono- and dinucleotide expansions, 42 and 37, respectively). The regular tricodon-encoded part of some chimeric peptides corresponds to standard human mitochondrial proteins (mono- and dinucleotide expansions, six (AT6, CytB, ND1, 2xND2, ND5) and one (ND1), respectively). Chimeric translation probably increases the diversity of mitogenome-encoded proteins, putatively producing functional proteins. These might result from translation by tRNAs with expanded anticodons, or from regular tricodon translation of RNAs where transcription/posttranscriptional edition systematically deleted mono- or dinucleotides after each trinucleotide. The pairwise matched combination of adjacent peptide parts translated from regular and expanded codons strengthens the hypothesis that translation of stretches of consecutive expanded codons occurs. Results indicate statistical translation producing distributions of alternative proteins. Genetic engineering should account for potential unexpected, unwanted secondary products.

## Graphical Abstract

**Figure:**
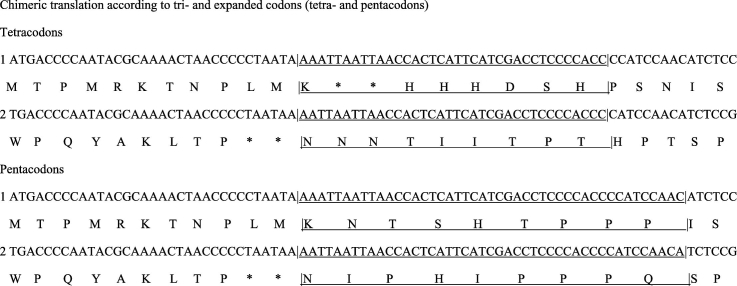

## 1 Introduction

The low stability of codon-anticodon duplexes does not enable mRNA translation without a ribosome stabilizing this interaction long enough to enable peptide elongation [1]. Ribosomes are very complex molecules [[2], [3], [4]] resulting from complex accretion histories [[5], [6], [7], [8], [9]], meaning that tRNA and tRNA-like structures accreted serially, becoming over time modern rRNA. A history derived from classical comparative analyses partly converges with an alternative, structure-based approach [[10], [11], [12], [13]], from tRNAs [10,[14], [15], [16], [17], [18], [19], [20]] and (perhaps) tRNA dimers [7,21,22]. Similar accretion histories also apparently produced other types of ancient (protein coding) genes [23]. Hence some kind of translation presumably occurred before ribosomes evolved, possibly including direct codon-amino acid interactions [[24], [25], [26], [27], [28], [29], [30], [31]].

### 1.1 Expanded Codons and Anticodons

Ribosome-free translation seems impossible because codon-anticodon duplexes are too weak to enable peptide elongation. A simple potential solution to ancestral ribosome-free translation by codon-anticodon interactions assumes that modern codons are reduced. Ancestral ribosome-free translation presumably resulted from interactions between codons and anticodons consisting of more than three nucleotides [1,[32], [33], [34]]. This stabilizes codon-anticodon duplexes long enough to enable ribosome-free peptide elongation (the ribosome also channels the spatial dynamics of aminoacylated tRNA-peptide interactions [35]). In addition, a code based on a specific subset of tetracodons (codons expanded by a fourth, silent nucleotide) would have self-correcting symmetry properties that could be an adequate ancestor of some modern (mitochondrial) genetic codes (the tessera hypothesis [36,37]).

Natural tRNA-based translation of tetracodons was observed from the onset of molecular biology [[38], [39], [40]]. Modern ribosomes accommodate tRNAs with expanded anticodons during translation [[41], [42], [43]]. Biotechnological applications use tRNAs with expanded anticodons to introduce non-natural amino acids in proteins [[44], [45], [46], [47], [48]]. The antisense sequence of some mitochondrial tRNAs has predicted expanded anticodons [49,50]. The predicted mitochondrial tRNAs with expanded anticodons coevolve with predicted mitochondrial tetracodon-encoded peptides [[51], [52], [53], [54]]. Tetracoding in modern organisms seems to be an adaptation to high temperatures where regular codon-anticodon interactions become relatively unstable: predicted tetracoding increases with lepidosaurian body temperature [55]. This would be a relict of prebiotic ribosome-free translation and genetic code formation, which likely occurred at high temperatures [[56], [57], [58], [59], [60]]; but see [61] for some counter-arguments to life's thermophilic origin hypothesis.

### 1.2 Peptides Coded by Expanded Codons

In addition to tetracoding sequences predicted by alignment methods, peptides corresponding in their entireties to the translation of tetra- and pentacodons have been detected in MS data (produced by the medium accuracy Thermo Fisher (Illkirch, France), and the high accuracy Waters (Milford, MA, USA) technologies) from the human mitochondrial peptidome [[62], [63], [64], [65], [66], [67]]. Detections of complete tetracoded peptides differ from occurrences of isolated tetracodons, notably in mitochondrial genomes [68,69], which might result from decoding by specific expanded anticodons or as regular codons after deletion of extra nucleotide(s) by post-transcriptional editing [70].

### 1.3 Alternative Translations

Translation according to expanded codons by a series of expanded anticodons is not the only known alternative translation. Antitermination, or stop suppression occurs due to decoding by regular near-cognate tRNAs [[71], [72], [73], [74], [75]] or tRNAs with an anticodon matching a stop codon [[76], [77], [78], [79], [80]]. Natural stop-suppressor tRNAs have also been adjusted for genetic code expansion to insert non-natural amino acids in proteins in biotechnological applications [[81], [82], [83]]. Natural stop suppression in mitogenes can be predicted based on alignment analyses [52,[84], [85], [86], [87], [88], [89], [90], [91]], and observed distributions of amino acids inserted at stops [[62], [63], [64], [65], [66], [67]] match genetic code evolution [[92], [93], [94], [95]] and coding symmetries in the genetic code [96].

### 1.4 Alternative Transcriptions

Post-transcriptional editing by systematically deleting every fourth, or every fourth and fifth nucleotide after each transcribed nucleotide triplet could produce noncanonical transcripts whose regular transcription matches noncanonical translation of regular transcripts according to tetra- or pentacodons. Mitochondrial transcripts detected in several independent datasets produced by independent sequencing technologies (Sanger and Illumina) match sequences predicted by systematic deletions after each transcribed nucleotide triplet. These noncanonical transcripts (delRNAs), because of their length corresponding to numerous tricodons separated by deleted mono- or dinucleotides, seem more likely produced by noncanonical transcription systematically deleting mono- or dinucleotides than by posttranscriptional edition, though the latter cannot be excluded [97]. These delRNAs have more than expected homopolymers [98] that frequently induce frameshifting [99,100].

Note that the human mitogenome, assuming systematic deletion of mono- or dinucleotides after each transcribed nucleotide triplet, includes more palindromes than random sequences with the same length and nucleotide content [[100], [101]].

A different type of noncanonical transcription exists, consisting of systematic exchanges between nucleotides, producing swinger RNAs that do not resemble their template DNA unless considering the transformation rule that produced the noncanonical RNA. Nine systematic nucleotide exchanges are symmetric (X<->Y, example G<->T [87,88,90], and fourteen are asymmetric (X->Y->*Z*->X, example C->T->G->C [89]). Empirical genomic coverages by swinger RNAs are replicable across independent datasets [98] and sequencing techniques (Sanger and Illumina, human mitogenomes [63]; 454 and SOLID, Mimivirus [102]). The human mitogenome, assuming systematic nucleotide exchanges, includes more palindromes forming stem-loop hairpins than random sequences with the same length and nucleotide contents [103]. Systematic nucleotide exchanges conserve error-correcting properties of the genetic code and its embedded circular code regulating ribosomal translation frame [[104], [105], [106], [107]].

### 1.5 Chimeric RNAs and Peptides

Note that swinger DNA has also been reported [108,109], in one case with abrupt switches between the regular and the swinger-transformed part of the mitogenome [110]. Chimeric RNAs, partly corresponding to regular transcription of the human mitogenome, and partly corresponding to swinger-transcription of adjacent parts of the mitogenome, also occur, including abrupt switches between these two parts [111]. These either result from regular transcription of genomic swinger DNA, or from swinger transcription of part of the template mitogenome. Chimeric peptides corresponding to translation of adjacent parts of regular and swinger-transformed RNA exist in mitochondrial proteomic data, including peptides whose regular part corresponds to mitochondrion-encoded proteins, for example CytB [112].

These chimeric molecules are strong evidence for swinger phenomena. First because if they reflected unknown artifacts, these would not have produced the regular parts of the RNA and peptide sequences. Chimeric RNAs and peptides show that unknown phenomena producing variants of known RNAs and proteins exist. In addition, the regular parts of the chimeric RNA/peptide are natural matched positive controls for adjacent noncanonical parts. This strengthens the confidence in the biological reality of these noncanonical phenomena.

**Fig. 1:**
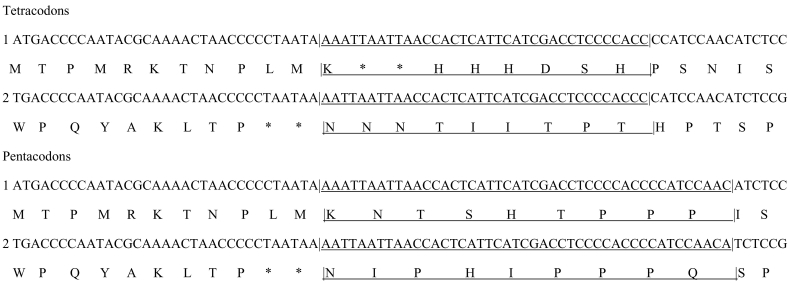
RNA sequence and its chimeric translation according to regular tricodons and tetra- and pentacodons. Sequences corresponding to 90 codons (two groups of 30 regular tricodons, each at the 5′ and 3′ extremity of a group of 30 noncanonical codons expanded by mono- or dinucleotides (tetra- and pentacodons)) form running windows of 90 + 120 + 90 = 300 nucleotides (tetracodons) and 90 + 150 + 90 = 330 nucleotides (pentacodons). Hence for each of the 16,569 positions along the human mitogenome, chimeric peptides are translated from 30 regular, 30 noncanonical and 30 regular codons. These hypothetical peptides (lengths truncated in Fig. 1 for presentation purposes) are compared with actual MS mitoproteomic data.

In the context of long stretches of translation according to expanded codons, chimeric peptides corresponding in part to regular translation, and in adjacent parts to translation according to expanded codons (Fig. 1), would also consist strong evidence for translation of stretches of expanded codons, and indicate that variants of known proteins including parts encoded by expanded codons exist. Hence here we present analyses of two mitochondrial MS datasets (one produced by Thermo Fisher and one by Waters technologies) that explore for chimeric peptides resulting from translation according to adjacent stretches of regular and expanded codons.

These analyses presume that translation produces a distribution of alternative protein products, some might present functional advantages, for example by having functional optima at conditions that differ from those of known canonical proteins (for example temperature). The approach implies caution: genetic engineering should account for potential unwanted byproducts resulting from little known and/or unknown alternative transcriptions and translations: unlike engineered genes, natural genes adapted to avoid or minimize disruptive effects by proteins resulting from rarer noncanonical transcriptions and/or translations.

## 2 Materials and Methods

The data and analytical methods are identical to those previously used for chimeric swinger peptides [112]. The human mitogenome (NC_012920, length 16,569 basepairs) was downloaded in its entirety.

### 2.1 Predicted Peptides

The difference with previous analyses consists in the fact that analyses compare observed MS/MS data with hypothetical peptides that result in part from canonical, and from noncanonical translations for consecutive, adjacent stretches of amino acids. The design of these hypothetical chimeric peptides uses two sizes of running windows: one for tetracodons (codons expanded by one nucleotide) and one for pentacodons (codons expanded by two nucleotides). Each running window codes for 30 amino acids according to regular tricodons, 30 consecutive amino acids coded according to noncanonical codons expanded by a mono- or dinucleotide, and another consecutive 30 amino acids coded according to regular tricodons. Hence each hypothetical chimeric peptide consists of 30 canonically coded amino acids at each its 5′ and 3′ extremities, and 30 noncanonical codons, each expanded by a mono- or dinucleotide. This produces window sizes of (3 × 30) + (4 × 30) + (3 × 30) = 90 + 120 + 90 = 300 nucleotides for tetracodons and 90 + 150 + 90 = 330 nucleotides for pentacodons. Windows move by steps of single nucleotides along the complete genome, producing 2 × 16569 theoretical chimeric tetra-, and 2 × 16569 chimeric pentacoded peptides, for + and − mitogenome strands.

For hypothetical chimeric peptides, the length of 30 consecutive amino acids with the same translation modus was chosen because previous experience shows that most detected peptides are shorter than 30 amino acids. Adopting shorter lengths, for example 15 amino acids, for the different parts of the hypothetical chimeric peptide, would not detect chimeric peptides with canonical and/or noncanonical parts longer than 15 amino acids.

For each hypothetical peptide, the relevant proteome analysis software predicts a theoretical mass spectrometry distribution. This distribution is compared with observed MS/MS data.

### 2.2 Translation of Stop Codons

Peptides translated from sequences including stop codons are included 19 times in the pool of theoretical peptides, each time inserting at all stop codons the same amino acid (there are 20 amino acids, but leucine and isoleucine have equal masses and hence cannot be distinguished by MS/MS techniques, resulting in 19 alternative peptides where a different amino acid species is inserted at all stops). Hence analyses consider the possibility that each amino acid can translate stop codons. Approximately 2 × 16569 × 19 = 629,622 chimeric peptides exist for each tetra- and pentacoded chimeric translations.

### 2.3 Medium Accuracy Data Searches

For the MS/MS data from [113], consensus searches between observed and predicted MS/MS data were handled with the Sequest (Thermo Fisher Scientific, Illkirch) algorithm with the following mass tolerances: Parent = 1 Da and Fragment = 0.5 Da (monoisotopic masses). Fixed carbamidomethyl (C) and variable Oxydation (M) modifications were activated, as well as the lysine → pyrrolysine modification, and only one missed trypsin cleavage was allowed. False discovery rate was estimated against a reverse decoy database using the Percolator algorithm. No protein grouping was allowed since the database only contained non-redundant entries. Peptides with false discovery rate q < 0.05 and score Xcorr >1.99 were considered identified. The score Xcorr is a likelihood of match between expected and observed MS/MS data unaffected by peptide length. The false discovery rate q is adapted to populations of detected peptides [114].

Previous analyses show that searches of the trypsinized proteome produced by [113] detect much more peptides when analyzing data separately for peptides cleaved at K, and separately for those cleaved at R, and when searches assume cleavage at the amino extremity of these amino acids rather than the carboxyl extremity at which trypsin cleaves proteic chains. These searches mainly detect peptides ending at the carboxyl extremity of K or R, corresponding to the experimental trypsin cleavage by assuming one missed cleavage. This increased efficiency of searches remains unexplained, but is not due to artifacts, because noncanonical peptides detected by all searches (assuming cleavage at any amino acid and at any (carboxyl and amino) extremities) map with comparable rates on corresponding, detected noncanonical RNAs, and correspond in their overwhelming majorities to trypsinized peptides as expected by the experimental parameters (addition of trypsin) [66].

### 2.4 High Accuracy Data Searches

The same pool of theoretical chimeric peptides was used for PLGS searches of another human mitoproteome dataset [115], extracted by a higher accuracy method (Waters, Milford, MA). Mass peak estimates are more accurate (5 ppm for data extracted by [115], versus 0.5 Da for data by [113]). Precise comparison of accuracies between these techniques is unfeasible: Sequest (Thermo Fisher Scientific, Illkirch) uses fixed cutoffs; PLGS adapts cutoffs to masses of detected peptide: 0.5 Da in the latter sample would occur for peptides with mass 5 × 10^6^ × 0.5 Da.

The twelve samples from [115] were processed using ProteinLynx Global Server version 3.0.1 (Waters, Saint-Quentin En Yvelines, France). Processing parameters were 250 counts for the low energy threshold, 100 counts for the elevated energy threshold and 750 counts for the intensity threshold. Hits are considered significant according to standard criteria, with PLGS peptide score 6.49. This score is compared to a decoy database to estimate FDR, as done for Xcorr from the dataset produced by [113], and peptides with q < 0.05 are retained.

Each sample was searched separately for peptides 38 times, each search assuming cleavage at a different extremity (carboxyl or amino) of each amino acid species (merging L and I, 2 × 19 = 38).

### 2.5 Minimal Size of Detected Chimeric Peptides

Detected peptides are further filtered so as to retain only peptides with at least eight consecutive amino acids coded according to regular codons, and at least eight consecutive amino acids coded according to noncanonical expanded codons. This size was determined so that each regular- and noncanonically-encoded parts of the chimeric peptide have an approximate maximal e value 0.0014 (629622 × 1/19^8^).

## 3 Results and Discussion

### 3.1 Chimeric Peptides With a Tetra- or Pentacoded Part

Tables 1 and 2 present 28 chimeric peptides detected in the MS/MS data published by [113] and 14 chimeric peptides detected in the MS/MS data published by [115], with at least eight amino acids coded by tricodons and eight adjacent amino acids coded by tetracodons. Tables 1 and 2 also present 19 chimeric peptides detected in the MS/MS data published by [113] and 18 chimeric peptides detected in the MS/MS data published by [115], with at least eight amino acids coded by tricodons and eight adjacent amino acids coded by pentacodons.

**Table 1:** Chimeric peptides transcribed in part according to regular, and in part according to expanded codons (tetra- and pentacodons) from the human mitogenome, detected in MS data from Guegneau et al. (2014). Columns are: 1. Regular tricodon translation frame (positive strand, 1–3; negative strand, 4–6), tetra- and pentacoded parts indicated by T and P; 2. Position of regular tricoded part on translated human mitogenome; 3. S, amino acid inserted at stop codons; 4. Detected peptide sequence, minor letters indicate translated stops, “|” separates regular tricoded from other part, underlined parts are tetra- and pentacoded. Ambiguous limits between tricoded and other part are also indicated when occurring, ambiguous part is considered parsimoniously as tricoded. 5. Xcorr between expected and observed MS; 6. PSM, counts observed MS matching expected MS; 7. q, false discovery rate; 8. PEP, posterior error probability, peptide specific; 9. Position-specific amino acid modifications; 10. Positions in regular mitogenome-encoded proteins matching regular tricoded part of detected chimeric peptide.

T	Pos	S	Peptide	Xcorr	PSM	q	PEP	Modifications	Gene
4	359–373	A	KGGaYISGA|aaSGENSVNVIKEaa	3.65	193	0	0.473		
5	2140–2155	D	KALENFGKGAAGDGRAHRdVIF|MdPLSCGSQNVMIISS	3.26	15	0	0.533	K8(Lys- > PyrLys); C28(Carbamidomethyl); M34(Oxidation)	
6	1648–1661	D	KSMQWAILGLFVVG|SGLFNILdEV	2.54	7	0	0.181	M3(Oxidation)	
4	527–537	D	RdDMSAWL|ddRMIQPdFTS	3.59	299	0	1		
3	1774–1783	F	RESKNMPISHIfH|ITLLNLYFYL	2.21	31	0	0.449		nd2 285–294
3	4102–4113	G	KNFGATPNKSNN|QQLgTPNLLPIPHLPPVTY	2.28	1	0	0.907		
5	4348–4356	G	RWCgGWWg|M|GGLGSWESLGS	3.87	221	0	0.942	C3(Carbamidomethyl)	
5	5417–5427	G	KRGgGGLVE|IFL|DSCEVLATSLYICL	3.13	1	0	0.513	C15(Carbamidomethyl); C25(Carbamidomethyl)	
2	378–397	X	RQNTTSHSLKLKGPGGASY|P|LiAVCIMTRQLPLCQLM	2.48	1	0	0.219	K10(Lys- > PyrLys); C25(Carbamidomethyl); C34(Carbamidomethyl)	
4	936–945	X	KWSiLEFGEGLCWi|G|CGGNVVSNE	2.36	10	0	0.744	C12(Carbamidomethyl); C16(Carbamidomethyl)	
1	1762–1780	X	KTMASSSPPSiPPSPSLT|S|LYiPITHSSTLPI	3.99	1	0	0.696		
6	2565–2578		KPMITVPAHKGMA|M|LVMMLVLCNS	2.13	27	0	1	K1(Lys- > PyrLys); C22(Carbamidomethyl)	
4	1702–1712	M	KSTAASTIDPA|mG|SNGLGAmWAE	4.18	286	0	0.532		
4	3436–3445	N	RPPLnQMRAG|EGGGnIKVSFL	3.84	81	0	0.735		
1	2883–2890	N	RLITTQQW|QnMTQKnYLPNnDD	2.72	3	0	0.226	K14(Lys- > PyrLys)	at6 41–48
6	1138–1148	P	KCVGQDMpI|W|ISGLFSApGW	4.41	542	0	1	C2(Carbamidomethyl); M7(Oxidation)	
1	3854–3862	R	rHNYNKLH|L|LHNNQIVMLP	3.45	17	0	0.551	M17(Oxidation)	
1	4324–4353	R	PLLGLLLAAAGKSAQLGLHPWLPSAMEG|PT|QLAYPSAYVr	2.08	2	0	1		nd5 223–241
4	564–575	T	KGVSVGtVMLDSLG|I|WtItQAPtSEP	4.14	2	0	1	M9(Oxidation)	
6	1058–1076	V	KVGGEWSMFDSLYFDI|C|SLLvLWMMDPEHMNSMAL	2.05	1	0	0.909	C17(Carbamidomethyl); M33(Oxidation)	
5	2583–2595	W	RYwDAwQVK|MVGWLVwMSEAGV	2.62	4	0	0.527		
3	5404–5433	Y	KPLPATAV|SNQPSTITHQLQLQSHPSPTyMPTNLPTLy	2.65	11	0	0.493		
4	2233–2247	Y	RRAWTKYVDEMNM|VG|GWSyyWGKLSQyW	2.18	2	0	0.684	K6(Lys- > PyrLys); M11(Oxidation); K23(Lys- > PyrLys)	
6	4397–4404	S	RSVSIsNA|MHWSDMSEGWHGSFsKDsLYLSLIYGY	2.6	1	0	0.288	M9(Oxidation)	
2	378–397	P	RQNTTSHSLKLKGPGGASY|P|LpAVCIMTRQLPLCQLM	2.36	1	0	0.319	K10(Lys- > PyrLys); C25(Carbamidomethyl); M27(Oxidation); C34(Carbamidomethyl)	
3	1909–1916		KLVTLAPMTAH|L|LLPPPGK	2	1	0	1	K1(Lys- > PyrLys); M8(Oxidation); K19(Lys- > PyrLys)	
5	4085–4096	K	kAPIIYSIKV|TL|FNNSWL	2.93	65	0	0.34	K1(Lys- > PyrLys)	
5	3393–3403	K	kLYCVWM|M|APKMEETPA	3.51	228	0	0.64	K1(Lys- > PyrLys); C4(Carbamidomethyl); M7(Oxidation); M8(Oxidation)	
P									
2	1441–1466	A	KMSAETDSMALT|LISaTMaIEPIPENPKFSVPPITPHP	2.29	1	0	0.338	K1(Lys- > PyrLys); M18(Oxidation)	
4	3294–3302	D	RSSKLQYGd|FPAVMNNSVRKEGWdWSS	2.92	69	0.046	0.257	K4(Lys- > PyrLys); M14(Oxidation); K20(Lys- > PyrLys)	
2	282–293	D	KFNdAMLTPGL|V|LWARSRNN	3.56	4	0	1	M6(Oxidation)	
4	4257–4264	E	KGGEVKGA|FeWISELVFMILLAQRMGSDWLPSGE	2.72	61	0	0.73	M25(Oxidation)	
4	460–473	G	KFVITVAPQNDIW|P|RGYgSVgLgEgPVSSVDDVMPPCGDg	2.44	2	0	0.412	C37(Carbamidomethyl)	
2	260–280	G	RKESQTAA|S|KRLAgPHPHGKQQWLTFSNK	3.53	1	0	0.838		
4	3176–3187	G	RMYgKDWgLLVAggKSMALMKQPW|G|HSGSGLQRSTC	2.58	48	0	0.865	K5(Lys- > PyrLys); K15(Lys- > PyrLys); C36(Carbamidomethyl)	
5	1645–1652	I	RNSGCECViGM|A|DWiVCNE	2.57	65	0	1	C5(Carbamidomethyl); C7(Carbamidomethyl); M11(Oxidation); C17(Carbamidomethyl)	
2	948–959	M	RAVHAKTSPVKA|MLQYHIAmKSREPLL	2.02	1	0	0.296		
6	3343–3360	M	KmmLMMVLPGRK|G|VEVAVCmmYSDASSmDWEmmE	2.18	1	0	0.774	C19(Carbamidomethyl)	
2	282–292	M	KFNmAMLTPGL|ESSDRSLTI	4	289	0	0.986		
2	282–292	N	KFNnAMLTPGL|ESSDRSLTI	3.46	80	0	0.531	M6(Oxidation)	
4	2272–2287	N	KWWSGPGQNCRIVKVG|TRSTLNLVGGNNNDPV	2.84	1	0	0.716	C10(Carbamidomethyl)	
5	1645–1652	Q	RNSGCECVqGM|A|DWqVCNE	2.11	9	0	1	C5(Carbamidomethyl); C7(Carbamidomethyl); C17(Carbamidomethyl)	
3	3879–3889	Y	KVNKAMHEyQTHYTYP|TYPSyyQPFSS	2.33	4	0	0.388		
4	4257–4264	I	KGGEVKG|A|FiWISELVFMILLAQRMGSDWLPSGE	2.64	65	0	0.618	M18(Oxidation); M25(Oxidation)	
5	1645–1652	K	RNSGCECVkGM|A|DWkVCNE	2.11	10	0	1	C5(Carbamidomethyl); C7(Carbamidomethyl); C17(Carbamidomethyl)	
3	5166–5176	K	KQTIQDP|A|TQTIMPkPTP	2.98	128	0	0.538	K1(Lys- > PyrLys); K15(Lys- > PyrLys)	
2	3153–3162	K	RKHQPTPCKGSTI|P|IYYLKSFFL	2.44	1	0	0.307	C8(Carbamidomethyl); K9(Lys- > PyrLys)

**Table 2:** Chimeric peptides transcribed in part according to regular, and in part according to expanded codons (tetra- and pentacodons) from the human mitogenome, detected in MS data from Alberio et al. (2014). PLGS is the score estimating goodness of fit between observed and expected MS in the PLGS peptide detection software. Δ ppm is the difference between expected and observed MS total mass. Cl indicates cleavage expected by the MS/MS search that detected the specified peptide, C indicates cleavage at the carboxyl-, and N the amino-end of the amino acid. Chym and elas indicate cleavage by chymotrypsin and elastase.

T	Pos	S	Peptide	PLGS	PSM	Δ ppm	Gene	Cl
			Tetracoding					
3	4428–4435	T	NRHQPTTP|TSSLLPPStTNF	6.79	48	−2.7085		Chym
2	179–187	E	HNQPAIYQTTTLe|PYP|EPTKPQe	6.51	41	−4.7043		Hn
2	1612–1620	E	HSSPeYQA|P|SDIRPASS	6.51	37	−6.2392		Hn
1	1340–1352	D	SHANHNLYMTP|TTTIFLGTTYDAL	6.54	41	1.9161	ND1, 234–250	Sn
5	3210–3220	H	KMNPhAQSTA|A|IFMCSWVGSS	6.49	54	−3.1235		Kn
1	2296–2304	E	TEAMWNDL|L|eLDPGSLLSRGADGFMA	6.64	41	−0.7789		Tn
5	4157–4165	I	CRFiNGGI|VG|SWWQNML	6.79	38	2.4082		Cn
2	3525–3531	R	YTLSPMSW|r|NNTIAVH	6.74	22	−2.4967		Yn
3	1639–1658	Q	NVSLLLTLSILSIMAGSWGG|QPTSTKQYP	6.56	39	−1.0766	ND2, 150–169	Nn
1	1769–1779	N	AFPNGISnFQKnAHI|P|PSnPPSPSLT	6.59	58	1.8674		Tc
1	5433–5443	Q	TTGTTTTT|L|TVHSTqSHLP	6.72	31	0.5116		Pc
2	249–264	P	pSHLNHTS|kEQASSTQQCSSkRLA	6.83	39	−1.917		Pn
2	5240–5248	R	YNPSLT|qTFPqPqTAH	6.75	74	1.0238	CytB, 325–333	Yn
1	2182–2197	K	YWLLAADL|L|FNWSrHHN	6.66	35	−0.3615		Yn
			Pentacoding					
1	853–861	S	FPCTKSSQPMsPCs|HV|sRPRYPN	6.6	33	−1.8371		Nc
3	5074–5082	F	TfFNESEEA|TVHPLTSTSSFLFAPQ	6.94	40	−2.8644		Qc
3	4612–4622	D	LSNSALSSNL|S|PdPQLPNQQT	6.49	58	0.6481		Tc
2	399–415	H	hGACSVIDKPRSTS|P|SLPMALAPMGQ	6.51	58	2.9743		Hn
1	3776–3783	Y	HHySKFLHSAyYN|L|SQQLNMT	6.47	35	0.8352		Tc
1	1287–1294	H	PDLAHPGh|W|FISTLAE	6.9	33	1.6579	ND1, 185–192	Ec
1	651–667	S	LCSKMVGsFMGsGDKP|T|AVSVPMISNS	7.02	63	−0.6754		Ln
5	1151–1158	A	WVaaFLL|Q|MASSGaGGLM	6.72	40	−1.7249		Wn
6	457–467	C	RMVSLcLLWPLcM|ISSGMVcGLF	6.84	23	−7.7922		Chym
2	3143–3154	F	QHTMNWRfRKHQPTP|CPKfPSMRDNPI	6.57	36	−0.9708		Elas
6	5476–5483	S	SLRVMSG|s|QESKTDTA	6.8	71	−6.8916		Ac
4	4033–4041	E	KICAAVECADeeDVAG|e|LVREGYNQ	6.53	29	0.04081		Qc
1	2017–2024	N	VTTTSTT|L|FSLLDTFSN	6.73	34	−2.3462		Nc
6	5475–5483	M	MLRVMMG|m|QESKTDTA	6.83	26	1.7577		Ac
2	925–933	Y	KNHGyYLHNHT|Q|VLNYQTCI	6.69	28	−3.8878		Kn
1	1122–1136	H	NAYRTKNShLYTTT|Q|ANWAHAHP	6.62	32	0.2141		Pc
4	2632–2643	G	DgSLLGGDgSVV|EDLGGKgDSEVAGGSWGMWRSF	6.63	31	−1.6834		Fc
2	4073–4081	Y	SQELTLYyA|Q|ELLTHAPM	6.83	38	−2.4583		Sn

In about half the cases, the noncanonical part of the peptide is on the 5′ extremity of the peptide, for tetra- and pentacoded parts, for any dataset. The noncanonical part can either be at the 5′- or the 3′-encoded extremity of chimeric peptides, with no apparent bias for one of these extremities in the current results.

### 3.2 Chimeric Peptides Integrated in Regular Mitogenome-Encoded Proteins

Most peptides include amino acids inserted at stop codons, in each regular-encoded and noncanonical parts. The regular-encoded part of a total of seven chimeric peptides corresponds to one among the 13 classical mitogenome-encoded proteins. Six among these have adjacent tetracoded parts, and one an adjacent pentacoded part. The proteins are: AT6, CytB, ND1, ND2 (two different peptides), ND5 (tetracoded) and ND1 (pentacoded). These small numbers do not enable to test whether biases exist in terms of which proteins tend to include more or less noncanonical parts, nor in relation to their position on the mitogenome, as these genes are scattered across the whole mitochondrial operon. The hypothesis that noncanonical peptides result from mitochondrial polymorphisms and heteroplasmy [[116], [117], [118], [119], [120], [121]] is unlikely: their exact correspondence to sequences predicted by translation of expanded codons excludes this option. As a group, they cannot result from regular mitogenomic DNA variability.

### 3.3 Noncanonical Transcription or Translation?

Above results confirm that tetra- and pentacoded of amino acid stretches occur, conjugated with regular encoded stretches of amino acids. The alternative, that chimeric peptides originate from regular translation of chimeric RNAs produced in part by regular transcription, and in part by noncanonical transcription systematically deleting mono- or dinucleotides after each transcribed trinucleotide, cannot be excluded. The data at hand don't enable to test between these two alternatives potentially producing identical peptides. We tentatively presume that both mechanisms are at work because expanded codons and anticodons have been previously reported, and because noncanonical transcripts corresponding to transcription systematically deleting mono- and dinucleotides also exist.

### 3.4 Adaptive Diversity

Amino acid stretches encoded by noncanonical codons (or resulting from noncanonical transcription) might be integrated in regular mitogenome-encoded proteins, as suggested by their association with stretches of tricodon-encoded amino acids clearly corresponding to regular membrane-bound mitochondrial proteins. The possibility that these chimeric peptides are part of functional proteins cannot be excluded. The existence of chimeric peptides suggests that natural protein diversity can be increased by mixing types of decoding processes, such as regular tricodons and noncanonical codons expanded by one or two nucleotides. This diversity might have unknown adaptive/functional components, including widening ranges of functionally optimal conditions at which some metabolic activities might occur.

Results also stress that natural translation of expanded codons is not extremely rare. The hypothesis that expanded codons (but not systematic deletions) are adaptive at high temperatures could be tested by comparing abundances of detected peptides coded by expanded codons at different temperatures, expecting more translation according to expanded codons at higher temperatures. Other analyses searching for peptides corresponding to codons expanded by more nucleotides (>2) will also contribute to our understanding of these noncanonical transcriptions and translations that increase the coding potential of sequences.

### 3.5 Unwanted Effects of Genetic Engineering

Experiments and analyses exploring for which genome regions undergo the different noncanonical transcriptions and translations in which cell types and under which conditions would deepen our understanding of cell metabolism, implying likely biomedical applications. Results also stress that genetic engineering should explore potential effects of proteins produced from noncanonical transcripts and/or by noncanonical translations, to avoid undesirable effects from discarded noncanonical processes such as swinger and del-transcriptions, and translation of stop codons and according to expanded codons.

## Declaration of Competing Interest

None.
